# Systematic Evaluation of the Patient-Reported Outcome (PRO) Content of Clinical Trial Protocols

**DOI:** 10.1371/journal.pone.0110229

**Published:** 2014-10-15

**Authors:** Derek Kyte, Helen Duffy, Benjamin Fletcher, Adrian Gheorghe, Rebecca Mercieca-Bebber, Madeleine King, Heather Draper, Jonathan Ives, Michael Brundage, Jane Blazeby, Melanie Calvert

**Affiliations:** 1 Primary Care and Clinical Sciences, University of Birmingham, Birmingham, United Kingdom; 2 The Nuffield Department of Primary Care Health Sciences, University of Oxford, Oxford, United Kingdom; 3 Department of Global Health and Development, London School of Hygiene & Tropical Medicine, London, United Kingdom; 4 Quality of Life Office, Psycho-oncology Co-operative Research Group, School of Psychology, University of Sydney, Sydney, Australia; 5 Medicine, Ethics, Society and History, University of Birmingham, Birmingham, United Kingdom; 6 Queens University, Kingston, Ontario, Canada; 7 Medical Research Council ConDuCT II Hub for Trials Methodology Research, School of Social & Community Medicine, University of Bristol, Bristol, United Kingdom; 8 MRC Midland Hub for Trials Methodology Research, University of Birmingham, Birmingham, United Kingdom; University Hospital Basel, Switzerland

## Abstract

**Background:**

Qualitative evidence suggests patient-reported outcome (PRO) information is frequently absent from clinical trial protocols, potentially leading to inconsistent PRO data collection and risking bias. Direct evidence regarding PRO trial protocol content is lacking. The aim of this study was to systematically evaluate the PRO-specific content of UK National Institute for Health Research (NIHR) Health Technology Assessment (HTA) programme trial protocols.

**Methods and Findings:**

We conducted an electronic search of the NIHR HTA programme database (inception to August 2013) for protocols describing a randomised controlled trial including a primary/secondary PRO. Two investigators independently reviewed the content of each protocol, using a specially constructed PRO-specific protocol checklist, alongside the ‘Standard Protocol Items: Recommendations for Interventional Trials’ (SPIRIT) checklist. Disagreements were resolved through discussion with a third investigator. 75 trial protocols were included in the analysis. Protocols included a mean of 32/51 (63%) SPIRIT recommendations (range 16–41, SD 5.62) and 11/33 (33%) PRO-specific items (range 4–18, SD 3.56). Over half (61%) of the PRO items were incomplete. Protocols containing a primary PRO included slightly more PRO checklist items (mean 14/33 (43%)). PRO protocol content was not associated with general protocol completeness; thus, protocols judged as relatively ‘complete’ using SPIRIT were still likely to have omitted a large proportion of PRO checklist items.

**Conclusions:**

The PRO components of HTA clinical trial protocols require improvement. Information on the PRO rationale/hypothesis, data collection methods, training and management was often absent. This low compliance is unsurprising; evidence shows existing PRO guidance for protocol developers remains difficult to access and lacks consistency. Study findings suggest there are a number of PRO protocol checklist items that are not fully addressed by the current SPIRIT statement. We therefore advocate the development of consensus-based supplementary guidelines, aimed at improving the completeness and quality of PRO content in clinical trial protocols.

## Introduction

The value of assessing patient-reported outcomes (PROs) in clinical trials has been emphasized by major international health-policy and regulatory authorities, and by patients [1]–[3]. PROs are increasingly selected as primary, secondary or exploratory outcomes within clinical trials as they provide the patient's perspective on the physical, functional and psychological consequences of treatment and the degree and impact of disease symptoms (Table 1) [4]. If captured in a scientifically rigorous way, PRO results may aid clinical decision-making [5], support labelling claims [6] and influence healthcare policy [7]. It is important, therefore, that details regarding PRO assessment are included in the trial protocol, to ensure that PRO data is collected and managed appropriately.

**Table 1:** Definitions.

Definitions:
Patient-Reported Outcome (PRO) – “… any report of the status of a patient's health condition that comes directly from the patient, without interpretation of the patient's response by a clinician or anyone else.” [6]
Patient-Reported Outcome (PRO) – “… any report of the status of a patient's health condition that comes directly from the patient, without interpretation of the patient's response by a clinician or anyone else.” [6]

The trial protocol is a key document, which should provide sufficient detail to facilitate understanding of the study design and administration, and enable appraisal of the trial's scientific, methodological and ethical rigor by funders and ethics committees [8], [9]. However, important information relating to study design, implementation and dissemination is often omitted from trial protocols [10]–[12]. This has led to the development of international guidance for protocol developers and reviewers, in the form of the SPIRIT 2013 statement (Standard Protocol Items: Recommendations for Interventional Trials), which is aimed at enhancing general study design, conduct, reporting and external review [8], [9]. PRO-specific information within trial protocols has received little scrutiny to-date, however, recent qualitative evidence suggests that it is sub-optimal [13]. This may lead to variations in PRO measurement across trial sites, potentially degrading data quality and biasing trial results [13]. Our objective was to systematically review randomised controlled trial (RCT) protocols including either a primary or secondary PRO outcome, evaluating the completeness of their PRO-specific content using a specially developed PRO protocol checklist. We also used the SPIRIT tool to measure how complete the protocols were in broad terms, to investigate whether levels of PRO content were associated with general protocol completeness.

## Methods

### Ethics

The University of Birmingham ethical review board approved this study (ERN_13-0047).

### Protocol Selection

We reviewed protocols submitted to the National Institute for Health Research (NIHR) Health Technology Assessment (HTA) programme, reasoning they would provide a representative snapshot of such documentation within the domain of health-care research. The NIHR-HTA programme is the largest such funding stream in the UK (comparable to the National Institutes of Health in the US and the Australian New Zealand Clinical Trials Registry in Australasia) and as a public interest funder, promotes the inclusion of patient-centred outcomes in its research [14]. Two investigators (BF, HDu) independently reviewed the NIHR-HTA database (inception to August 2013, http://www.hta.ac.uk/research/index.shtml) for RCTs with a primary or secondary PRO endpoint. Disagreements regarding trial eligibility were resolved through discussion with a third reviewer (DK/MC). The most up-to-date trial protocols were retrieved for review, either from the HTA database, the trial website, or via the named trial representative (contacted by email, followed by one email reminder after 2 weeks).

### Data Extraction

Two investigators (DK, HDu) independently extracted the following data from each protocol using a predesigned data extraction form: year of protocol publication, the name(s) of the PRO(s) used in the trial, whether the PRO was a primary or secondary outcome, the trial setting (primary or secondary care) and the clinical specialty.

### Protocol Checklists

**Figure 1:**
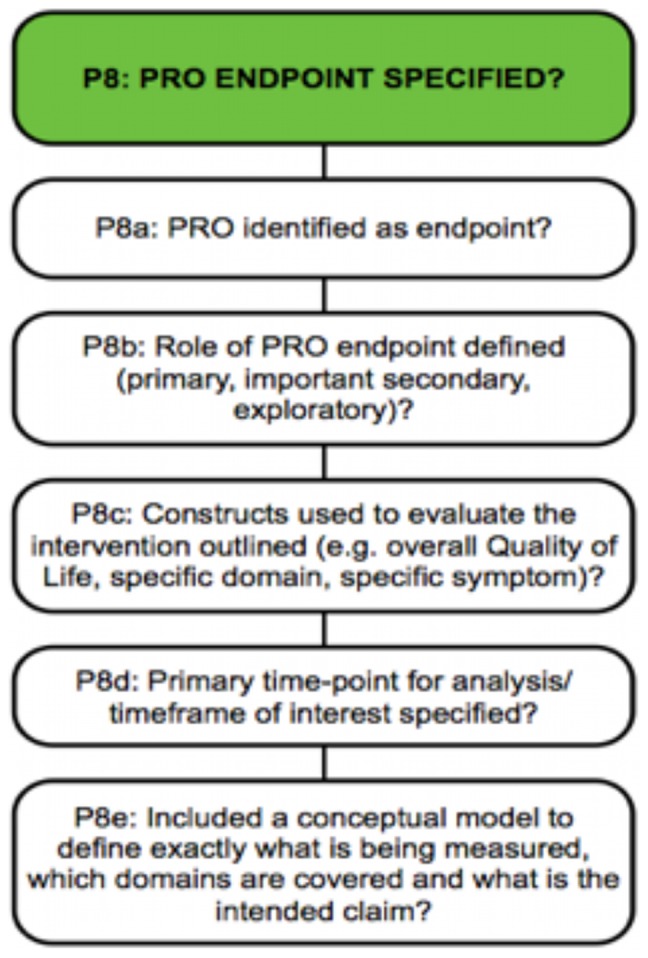
PRO protocol checklist item ‘P8’ and associated sub-categories.

The completeness of the PRO-specific content of trial protocols was assessed using a PRO protocol checklist (Table 2), generated from 162 recommendations identified in our systematic review of PRO-specific guidance for trial protocol writers [15]. Recommendations were grouped into major categories comprising 33 PRO-specific items for inclusion in a trial protocol. Individual recommendations were retained under each item as subcategories (illustrated in Figure 1). MC and DK constructed the initial framework of the PRO protocol checklist, which was then reviewed, amended where necessary, and subsequently approved by an international expert external advisory group (MB, JB, RMB, MK) (see Appendix S1 for the full checklist). The completeness of general sections within each protocol was assessed using SPIRIT, as a proxy measure of the overall strength of the protocol [8], [9]. The SPIRIT resources include a checklist [8] containing 51 individual recommended protocol items, spread over 33 categories and an accompanying explanatory paper [9] and website (www.spirit-statement.org).

**Table 2:** PRO-specific protocol checklist.

SPIRIT Section	Item Number	Description
**Administrative information**		
	P1	Roles & Responsibilities of PRO Personnel Identified?
**Introduction**		
	P2	Background PRO-specific information provided?
	P3	PRO-specific rationale provided?
	P4	PRO-specific hypothesis provided?
	P5	PRO-specific objectives stated (in relation to dimensions, population and timeframe)?
**Methods: Participants, interventions and outcomes**		
	P6	Details & rational of PRO study sample/setting provided?
	P7	PRO considerations discussed in the eligibility criteria?
	P8	PRO endpoint specified?
	P9	Timing of PRO assessments specified?
	P10	Timing of PRO assessments justified?
	P11	PRO sample size discussed & justified?
**Methods: Assignment of interventions (for controlled trials)**		
	P12	PROs discussed in relation to blinding?
**Methods: Data collection, management and analysis**		
	P13	PROM identified & described?
	P14	Choice of PROM justified in relation to study hypothesis?
	P15	Choice of PROM justified in relation to measurement properties?
	P16	Choice of PROM justified in relation to acceptability & patient burden?
	P17	PRO data collection plan included?
	P18	PRO data collection guidelines/training information provided for trial personnel?
	P19	Plans to minimise avoidable missing PRO data provided?
	P20	PRO-specific Quality Assurance (QA) described?
	P21	PRO Statistical Analysis Plan provided?
	P22	Plans to address multiplicity of PRO data provided?
	P23	PRO clinical significance defined?
	P24	Statistical methods to deal with missing PRO data defined?
**Monitoring**		
	P25	PRO data monitoring defined?
	P26	Plan for the identification and management of PRO Alerts included?
**Ethics and dissemination**		
	P27	PRO-specific consent information provided?
	P28	PRO-specific confidentiality procedures described?
	P29	PRO dissemination policy outlined?
**Appendices**		
	P30	PRO information included in consent materials?
	P31	PRO assessment checklist and/or flowsheet provided in appendix?
	P32	Exact version of PROM provided in CRF/appendix (with translated versions if appropriate)?
	P33	PROM completion instructions provided in CRF/appendix?

Abbreviations: SPIRIT, Standard Protocol Items: Recommendations for Interventional Trials; PRO, patient-reported outcome; PROM, patient-reported outcome measure; CRF, case report form.

### Protocol Review

Two investigators (DK, HDu) independently assessed the content of the included protocols using the PRO and SPIRIT checklists. For each trial protocol assessed, items on each checklist were either described as ‘present’ or ‘absent’. One point was assigned for each item ‘present’, giving a total score (maximum achievable, 51 for SPIRIT and 33 for the PRO checklist). In addition, for the PRO protocol checklist, the investigators also determined whether all sub-categories were satisfied for each item categorized as ‘present’. Therefore, PRO items that were marked as ‘present’, but that failed to satisfy all of the appropriate sub-categories were additionally tagged as ‘incomplete’. Levels of investigator agreement were determined for both checklists. Disagreements were resolved through discussion with a third investigator (MC) if required.

### Data Analysis

Analyses were performed using SAS V9.2 (SAS Institute, Cary NC). Descriptive analyses were conducted on the number of PRO-specific and SPIRIT checklist items present in the included protocols. To explore factors associated with the inclusion of PRO-specific protocol items, we performed a pre-specified multiple regression analysis in which the dependent variable was the PRO-specific protocol checklist score and the independent variables were: whether the PRO was named as a primary or secondary outcome, the trial setting, the clinical specialty and the SPIRIT checklist score. 75 protocols were required to satisfy the sample size requirement for this regression analysis (15 per co-variate [16]). The relationship between the PRO-specific protocol checklist score and the candidate explanatory variables was assessed using a backward stepwise selection process with α = 0.05 as criteria for model inclusion.

## Results

At the time of the review (August 2013) 459 studies were listed on the HTA database, of which 284 fulfilled the inclusion criteria. As our sample size requirement was 75, we restricted our review to the 75 most recent trial protocols to provide an up-to-date picture of the PRO-specific content in such documentation. Levels of investigator agreement for both checklists were high (85.77% for SPIRIT and 86.11% for the PRO checklist) and all disagreements were resolved through discussion. Characteristics of the included protocols are presented in Table 3. A PRO was the primary outcome in 41%; 38% were conducted in a primary care setting, 51% were conducted in secondary care and 11% were conducted in both. In total, 251 different PRO measures were used across the included trials (Appendix S2), the most common being the five dimension European Quality of Life instrument (EQ-5D), the Short-Form Health Survey 12-item (SF-12) and 36-item (SF-36) questionnaires and the Hospital Anxiety and Depression Scale (HADS).

**Table 3:** Characteristics of included protocols (N = 75).

Characteristic	Protocols, No. (%)
**Year**	
2012	29 (39)
2013	46 (61)
**Study PRO endpoint & setting**	
PRO 1° Outcome	31 (41)
Primary care setting	29 (38)
Secondary care setting	38 (51)
Both primary & secondary care	8 (11)
**Clinical Research Area**	
Mental Health	15 (20)
Neurology	8 (11)
Orthopaedics; Paediatric; Vascular	5 (7)
Obstetrics & Gynaecology; Oncology; Respiratory;	4 (5)
Cardiology; Physical Activity; Smoking Cessation	3 (4)
Falls Prevention; Gastroenterology; Weight Loss	2 (3)
Aids; Colorectal; Dermatology; Diabetes; Elderly Care; Emergency Services; General Practice; Hepatology; Nephrology; Urology	1 (1)
**PROMS** #	
European QOL instrument (EQ-5D)	56 (75)
Short-Form Health Survey 36-item (SF-36)	13 (17)
Short-Form Health Survey 36-item (SF-12)	12 (16)
Hospital Anxiety and Depression Scale (HADS)	9 (12)
Patient Health Questionnaire 9 (PHQ-9)	6 (8)
Pediatric Quality of Life Inventory (PEDSQL);	5 (7)
Epworth Sleepiness Scale (ESS); Beck Depression Inventory (BDI); Generalised Anxiety Disorder (GAD-7); Calgary Sleep Apnoea Quality of Life Index (SAQLI); Carer/Proxy/Parent Completion EQ-5D	3 (4)
Client Services Receipt Inventory (CSRI); WHOQOL-BREF Secondary; The Lubben Social Network Scale (LSNS); Resource Use questionnaire; Morisky Medication Adherence Scale; International Physical Activity Questionnaire (IPAQ); Clinical Outcomes in Routine Evaluation Outcome Measure (CORE-OM); Falls Efficacy Scale; Nottingham Activities of Daily Living (NEADL); Olerud & Molander Ankle Score (OMAS)	2 (3)

# PROMS listed used in >1 protocol. Total Number of PROMS used n = 251. A full list appears in Appendix S2. Abbreviations: PRO, patient-reported outcome; PROM, patient-reported outcome measure; QOL, quality of life.

### Adherence to SPIRIT and PRO Checklists

**Figure 2:**
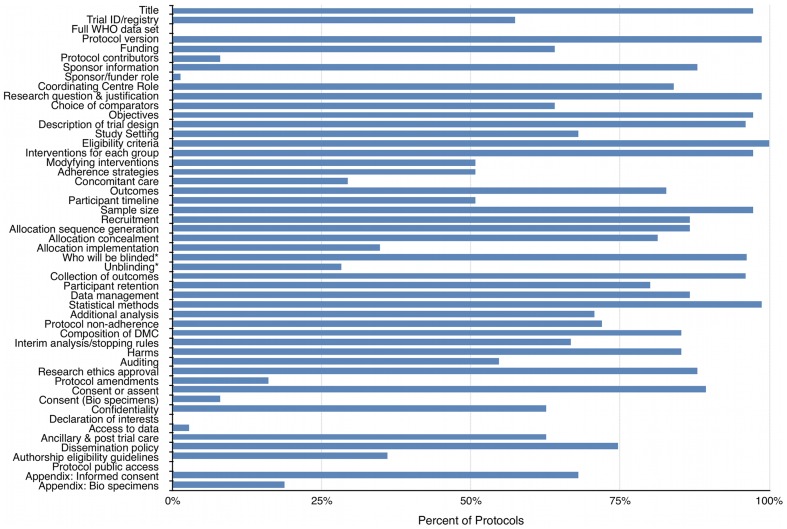
Protocol adherence to individual SPIRIT items. *Denominator adjusted as n = 46 blinded trials included in sample.

**Figure 3:**
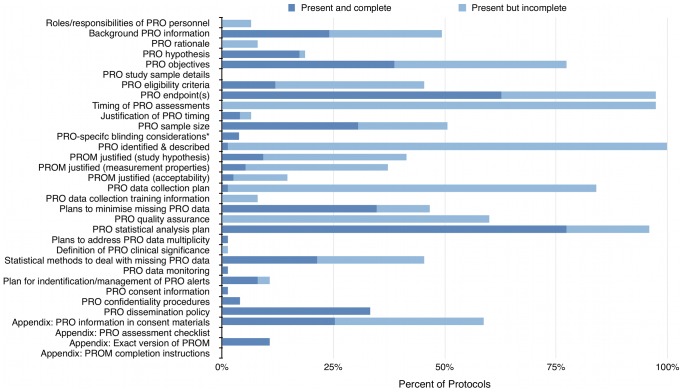
Protocol adherence to individual PRO items. *Denominator adjusted as n = 46 blinded trials included in sample.

Protocols included a mean of 32/51 (63%) SPIRIT recommendations (range 16–41, SD 5.62) and 11/33 (33%) PRO-specific items (range 4–18, SD 3.56). Protocol adherence to individual SPIRIT and PRO checklist items is presented in Figures 2 and 3, summarized in Table 4, and discussed below.

**Table 4:** Protocol adherence to individual SPIRIT and PRO checklist items (Sample, n = 75).

SPIRIT CHECKLIST	TOTAL	PRO CHECKLIST	COMPLETE	INCOMPLETE	TOTAL
**Administrative Information**					
ITEM 1: Title	**97.33%**				
ITEM 2A: Trial identifier and registry name	**57.33%**				
ITEM 2B: WHO Trial Registration Data Set	**0.00%**				
ITEM 3: Protocol version	**98.67%**				
ITEM 4: Funding	**64.00%**				
ITEM 5A: Protocol contributors	**8.00%**				
ITEM 5B: Trial sponsor information	**88.00%**				
ITEM 5C: Role of sponsor and funders in study	**1.33%**				
ITEM 5D: Roles of coordinating centre/steering committee etc.	**84.00%**				
		ITEM 1: Roles & Responsibilities of PRO personnel identified?	**0.00%**	**6.67%**	**6.67%**
**INTRODUCTION**					
ITEM 6A: Description of research question and justification for undertaking the trial	**98.67%**				
ITEM 6B: Explanation for choice of comparators	**64.00%**				
		ITEM 2: Background PRO-specific information provided?	**24.00%**	**25.33%**	**49.33%**
		ITEM 3: PRO-specific rationale provided?	**0.00%**	**8.00%**	**8.00%**
ITEM 7: Objectives	**97.33%**				
		ITEM 4: PRO-specific hypothesis provided?	**17.33%**	**1.33%**	**18.67%**
		ITEM 5: PRO-specific objectives stated (in relation to dimensions, population and timeframe)?	**38.67%**	**38.67%**	**77.33%**
ITEM 8: Description of trial design	**96.00%**				
**Methods: Participants, Interventions and Outcomes**					
ITEM 9: Study setting	**68.00%**				
		ITEM 6: Details & rationale of PRO study sample/setting provided?	**0.00%**	**0.00%**	**0.00%**
ITEM 10: Eligibility criteria	**100.00%**				
		ITEM 7: PRO considerations discussed in the eligibility criteria?	**12.00%**	**33.33%**	**45.33%**
**INTERVENTION**					
ITEM 11A: Interventions for each group	**97.33%**				
ITEM 11B: Criteria for discontinuing or modifying allocated interventions	**50.67%**				
ITEM 11C: Strategies to improve adherence to intervention protocols	**50.67%**				
ITEM 11D: Relevant concomitant care and interventions	**29.33%**				
ITEM 12: Outcomes	**82.67%**				
		ITEM 8: PRO endpoint specified?	**62.67%**	**34.67%**	**97.33%**
ITEM 13: Participant timeline	**50.67%**				
		ITEM 9: Timing of PRO assessments specified?	**0.00%**	**97.33%**	**97.33%**
		ITEM 10: Timings of PRO assessments justified?	**4.00%**	**2.67%**	**6.67%**
ITEM 14: Sample size	**97.33%**				
		ITEM 11: PRO sample size discussed & justified?	**30.67%**	**20.00%**	**50.67%**
ITEM 15: Recruitment	**86.67%**				
**Methods: Assignment of interventions (for controlled trials)**					
ITEM 16A: Allocation Sequence generation	**86.67%**				
ITEM 16B: Allocation concealment	**81.33%**				
ITEM 16C: Allocation Implementation	**34.67%**				
**BLINDING**					
ITEM 17A: Who will be blinded after assignment to interventions*	**96.23%**				
ITEM 17B: circumstances under which unblinding is permissible*	**28.30%**				
		ITEM 12: PROs discussed in relation to blinding?*	**3.77%**	**0.00%**	**3.77%**
**Methods: Data Collection, Management and Analysis**					
ITEM 18A: Plans for assessment and collection of outcomes	**96.00%**				
		ITEM 13: PROM identified & described?	**1.33%**	**98.67%**	**100.00%**
		ITEM 14: Choice of PROM justified in relation to study hypothesis?	**9.33%**	**32.00%**	**41.33%**
		ITEM 15: Choice of PROM justified in relation to measurement properties?	**5.33%**	**32.00%**	**37.33%**
		ITEM 16: Choice of PROM justified in relation to acceptability & patient burden?	**2.67%**	**12.00%**	**14.67%**
		ITEM 17: PRO data collection plan included?	**1.33%**	**82.67%**	**84.00%**
		ITEM 18: PRO data collection guidelines/training information provided for trial personnel?	**0.00%**	**8.00%**	**8.00%**
		ITEM 19: Plans to minimise avoidable missing PRO data provided?	**34.67%**	**12.00%**	**46.67%**
ITEM 18B: Plans to promote participant retention	**80.00%**				
ITEM 19: Data management	**86.67%**				
		ITEM 20: PRO-specific Quality Assurance (QA) described?	**0.00%**	**60.00%**	**60.00%**
ITEM 20A: Statistical methods for analysing primary and secondary outcomes	**98.67%**				
		ITEM 21: PRO Statistical Analysis Plan provided?	**77.33%**	**18.67%**	**96.00%**
		ITEM 22: Plans to address multiplicity of PRO data provided?	**1.33%**	**0.00%**	**1.33%**
		ITEM 23: PRO clinical significance defined?	**0.00%**	**1.33%**	**1.33%**
		ITEM 24: Statistical methods to deal with missing PRO data defined?	**21.33%**	**24.00%**	**45.33%**
ITEM 20B: Methods for any additional analyses (e.g., subgroup and adjusted analyses)	**70.67%**				
ITEM 20C: analysis population relating to protocol non-adherence	**72.00%**				
**MONITORING**					
ITEM 21A: Composition of Data Monitoring Committee (DMC) etc.	**85.33%**				
ITEM 21B: Description of any interim analyses and stopping guidelines	**66.67%**				
		ITEM 25: PRO data monitoring defined?	**1.33%**	**0.00%**	**1.33%**
ITEM 22: Harms	**85.33%**				
		ITEM 26: Plan for the identification and management of PRO alerts included?	**8.00%**	**2.67%**	**10.67%**
ITEM 23: auditing	**54.67%**				
**Ethics and Dissemination**					
ITEM 24: research ethics approval	**88.00%**				
ITEM 25: protocol amendments	**16.00%**				
ITEM 26A: consent or assent	**89.33%**				
ITEM 26B: consent or assent (Biological specimens)	**8.00%**				
		ITEM 27: PRO-specific consent information provided?	**1.33%**	**0.00%**	**1.33%**
ITEM 27: Confidentiality	**62.67%**				
		ITEM 28: PRO-specific confidentiality procedures described?	**4.00%**	**0.00%**	**4.00%**
ITEM 28: Declaration of interests	**0.00%**				
ITEM 29: Access to data	**2.67%**				
ITEM 30: Ancillary and post-trial care	**62.67%**				
ITEM 31A: Dissemination policy	**74.67%**				
ITEM 31B: Authorship eligibility guidelines	**36.00%**				
		ITEM 29: PROs dissemination policy outlined?	**33.33%**	**0.00%**	**33.33%**
ITEM 31C: Plans, if any, for granting public access to the full protocol	**0.00%**				
**APPENDICES**					
ITEM 32: Informed consent materials	**68.00%**				
		ITEM 30: PRO information included in consent materials?	**25.33%**	**33.33%**	**58.67%**
ITEM 33: Biological specimens	**18.67%**				
		ITEM 31: PRO assessment checklist and/or flowsheet provided in appendix?	**0.00%**	**0.00%**	**0.00%**
		ITEM 32: Exact version of PROM provided in CRF/appendix (with translated versions if appropriate)?	**10.67%**	**0.00%**	**10.67%**
		ITEM 33: PROM completion instructions provided in CRF/appendix?	**0.00%**	**0.00%**	**0.00%**

*Note: n = 46 blinded trials included in final sample, denominator adjusted accordingly. Abbreviations: SPIRIT, Standard Protocol Items: Recommendations for Interventional Trials; PRO, patient-reported outcome; PROM, patient-reported outcome measure; CRF, case report form; WHO, World Health Organisation.

### Administrative information

#### SPIRIT

Protocols routinely included general administrative information including: the project title (97% of protocols), protocol version (99%), trial sponsor (88%) and coordinating centre/steering committee details (84%). Just under two-thirds presented information regarding trial registration (57%) or sources of funding (64%). Few (8%) made it clear who had contributed to the production of the protocol.

#### PRO-specific

Five protocols (7%) included administrative information regarding the roles and responsibilities of trial personnel involved in the design and collection of PRO data.

### Introduction

#### SPIRIT

Almost all protocols (99%) included general background information in the introduction and outlined the trial rationale or included specific trial objectives or hypotheses (97%).

#### PRO-specific

Just under half of the protocols (49%) provided background details regarding the relevant existing PRO research (or lack of) in the area of interest, but very few (8%) included a rationale for the collection of PRO data within the trial. Over two-thirds also included PRO-specific objectives (77%), however, over one-third of these (39%) were incomplete, for example, details regarding the PRO dimensions under investigation or the timeframe of interest were often missing. In addition, less than one-third of protocols (19%) provided a PRO-specific hypothesis.

### Methods: Participants, Interventions and Outcomes

#### SPIRIT

Just over two-thirds of protocols (68%) included a description of the study setting(s), whilst 100% included general eligibility criteria. Protocols routinely included information on trial recruitment methods (87%), interventions (97%), outcomes (83%) and sample size requirements (97%). Half of the protocols (50%) presented criteria for discontinuing or modifying interventions, strategies to improve adherence to intervention protocols and included a participant time schedule. Less than one-third (29%) discussed relevant concomitant care and interventions.

#### PRO-specific

Just under half of the included protocols (45%) discussed PRO-specific eligibility considerations. None provided a description/rationale addressing which trial participants were eligible for PRO analysis. There was routine reporting of the timing of PRO assessments (97%), but justification for PRO timings was rarely provided (7%). PRO endpoints were described in nearly all protocols (97%), however, in more than one-third (35%) the information provided was incomplete, for example, the primary time-point for analysis, or an outline of the constructs used to evaluate the intervention (e.g. overall quality of life, or a specific domain/symptom) were frequently absent. Similarly, whilst PRO sample size requirements were provided in approximately half of the included protocols (51%), 20% of these failed to justify the assumptions of PRO analyses outlined.

### Methods: Assignment of Interventions (for controlled trials)

#### SPIRIT

All of the included trials were controlled and 61% employed some form of blinding. Most protocols detailed methods of allocation sequence generation and concealment (87% and 81% respectively), but few outlined who would assign participants to interventions (35%). Almost all protocols (96%) identified who would be blinded to the trial interventions, but less than one-third (28%) discussed the circumstances under which un-blinding was permissible.

### Methods: Data Collection

#### SPIRIT

Most protocols (96%) provided general plans for the assessment and collection of trial outcomes and over two-thirds (80%) described proposed strategies for the promotion of participant retention.

#### PRO-specific

PRO measures (PROMs) were always named (100%), but details regarding the measures were frequently missing, for example, the number of items/domains, methods for instrument scaling/scoring and estimated average completion time. The choice of PROM was rarely justified, whether in relation to the study hypothesis (justified in 41% of protocols), measurement properties (justified in 37%), or in relation to participant acceptability/burden (justified in 15%). Where some justification (of any type) was present (n = 33 protocols, 44%), it was commonly incomplete, for example, often information was not provided regarding the evidence-base (or lack of) for all measurement properties for a given tool, or for all tools used within a trial, and references were regularly absent. Brief information surrounding the plans for PRO data collection was included in 84% of protocols, but again elements were often absent, for example, there was a lack of information on who should administer the PROM and the level of assistance allowed during assessment, whether proxy assessment was permissible and where PRO assessment would take place. Just under half of the protocols (47%) detailed plans to minimize levels of avoidable missing PRO data. Finally, only 8% of protocols provided information surrounding PRO data collection guidelines and/or training for trial personnel.

### Methods: Management and Analysis

#### SPIRIT

Data management issues were discussed in 87% of protocols. Statistical methods for analysing (non-PRO) primary and secondary outcomes were routinely included in almost all (99%) protocols and over two-thirds discussed methods of additional analysis (71%) (e.g. subgroup analysis) and the handling of protocol non-adherence (72%).

#### PRO-specific

PRO-specific quality assurance issues were discussed in 60% of protocols. A PRO statistical analysis plan was provided in 96% of protocols, however, very few (1%) provided plans to address multiplicity of PRO data or were explicit about PRO clinical significance levels; and less than half (45%) detailed statistical methods to deal with missing PRO data.

### Monitoring

#### SPIRIT

Information regarding the Data Monitoring Committee, interim analysis, stopping guidelines and trial auditing arrangements was included in 85%, 67% and 55% of protocols respectively. Plans for monitoring and managing adverse events/harms were included in 85% of protocols.

#### PRO-specific

PRO-specific data monitoring issues were discussed in 1% of protocols. Plans for the identification and management of ‘PRO Alerts’ - where trial personnel encounter ‘concerning’ individual participant PRO data that may require a prompt response [17] - were included in 11% of protocols.

### Ethics and Dissemination

#### SPIRIT

Inclusion of ethics approval information (88%), informed consent/assent procedures (89%) and a dissemination policy (75%) was common. Just under two-thirds of protocols discussed confidentiality (63%) and ancillary and post-trial care (63%). There was, however, little consideration of authorship eligibility (36%), access to trial data (3%) or declaration of interests (0%).

#### PRO-specific

A third of protocols discussed PRO-specific dissemination (33%), but few (1%) tackled PRO consent or confidentiality issues.

### Appendices

#### SPIRIT

Fifty-one (68%) of the included protocols included patient information and consent materials in an appendix.

#### PRO-specific

PRO-specific information was included in 59% of patient information sheets. An exact version of the PROM(s) employed by the study was included in 11% of appendices; none included a PRO assessment checklist/flowchart.

### Determinants of Differences in PRO-specific Protocol Content

Table 5 summarizes the findings from our exploratory multiple regression analysis, which investigated predictors of differences in the PRO-specific checklist score between protocols. In the final model, only the nature of the PRO endpoint (primary versus secondary) was significant (P<.001), suggesting that protocols describing trials with a primary PRO include on average 5.00 (95% CI 3.79 to 6.21) additional recommended PRO-specific items compared to those employing a secondary PRO endpoint. There were no significant associations between the PRO checklist score and the year of protocol publication (P = .18), the trial setting (P = .08), the clinical specialty (P = .14) or the SPIRIT checklist score (P = .17). The full (first) model is presented in Appendix S3.

**Table 5:** Regression model investigating predictors of PRO-specific checklist score.^a^

Independent Variable	β (95% CI)	*P* Value	R^2^
PRO listed as the primary outcome	5.00 (3.79 to 6.21)^b^	<0.001	0.47^c^

Abbreviations: CI, confidence interval.

a Model with PRO protocol checklist score (max 33) the dependent variable (n = 75 included protocols).

b Intercept: 14.07 (95% CI 13.12 to 15.02).

c Reflects the proportion of variability in the PRO-specific checklist score explained by the statistical model.

## Discussion

### Summary of Findings

To our knowledge, this is the first study to evaluate the PRO-specific content of trial protocols. We found that routine inclusion of PRO information was poor (33%) and that over half (61%) of included PRO items were incomplete. Trials with a primary PRO endpoint tended to routinely include slightly more PRO information in their protocols (mean 43%). PRO protocol content was not associated with general protocol completeness; thus, protocols judged as relatively ‘complete’ using SPIRIT were still likely to have omitted a large proportion of PRO checklist items.

Our findings are concordant with the prevailing empirical evidence that important general methodological details are often missing from protocols [10]–[12], [18], [19]. On average, the reviewed protocols failed to include over one-third (37%) of the recommended protocol items outlined in SPIRIT [8] and over two-thirds (67%) of PRO checklist items. Our results also concur with qualitative data drawn from UK-based trial personnel, suggesting a widespread lack of PRO-specific information in clinical trial protocols and training [13].

Omission of recommended PRO content in trial protocols could lead to inconsistent assessment of important patient-centred outcomes [13], risking biased and unreliable trial results, and lessening the impact of PROs on routine clinical care. This practice may mislead clinical or health policy decision-making, reduce the value of patient participation in trials and waste limited healthcare and research resources: this is unethical [20].

The particularly low PRO checklist compliance we observed in our study is unsurprising, as evidence suggests existing PRO guidance for protocol writers is difficult to access and lacks consistency [15]. Until such time as this guidance improves, it may be difficult for researchers to effectively incorporate PRO information into their protocols. Unfortunately, our findings also suggest that PRO-specific protocol items are either not addressed by the current SPIRIT checklist (for example, the management of ‘PRO Alerts’ [17]), or are addressed only partially, such that fuller explanation is warranted to provide meaningful guidance to protocol developers who may not be familiar with PRO methodology (for example, approaches to minimise avoidable missing PRO data). The scope and number of additional PRO items, and the current lack of coherence in the guidance literature, justifies the need for supplementary PRO-specific guidelines. The PRO protocol checklist developed for this study could be incorporated into such guidelines. It is important to note, however, in designing the PRO checklist we deliberately sought to retain all PRO protocol guidance extracted in our review [15], without making a judgment on which items might be essential and which may be optional, or if the essential versus optional items might differ depending on whether a PRO was a primary or secondary outcome. The checklist therefore provides the research community with a comprehensive starting point, as opposed to a definitive tool; and does not amount to an international consensus, but rather represents an approximation of it for illustrative purposes. The next step would be for the PRO protocol checklist be subjected to a formal international consensus process to ensure that it provides appropriate and consistent guidance to protocol developers and focuses on only those PRO-specific protocol items that are deemed most important by the scientific community and other relevant stakeholders, including patients. Following this process, the checklist may prove a valuable addition to formal PRO protocol guidelines, aimed at improving the completeness and quality of PRO content in clinical trial protocols.

### Strengths and Weaknesses

The major strength of this study is its use of systematic methods and multiple reviewers at all stages. The SPIRIT 2013 statement was developed with comprehensive stakeholder involvement using rigorous and systematic methodology [21]. The PRO-specific checklist used in this study was developed by experts in the field, is supported by a systematic review of existing guidance [15] and demonstrated high levels of inter-rater agreement, however, it is yet to undergo a formal consensus process or validation. Both the PRO and SPIRIT checklists are still very recent and would not have been available to the developers of many of the included protocols, therefore validation of our findings in a contemporary sample of protocols is required. Our protocol sample is relatively small, and all describe trials that are UK-led (within a single funding stream), restricting generalizability. Nevertheless, the sample includes studies focusing on a range of clinical specialties, conducted in a variety of healthcare settings and employing a broad spectrum of PROs, thus enhancing external validity. Finally, it is possible that the trial protocols from other funding bodies are more advanced, in PRO terms, than those included in our review; although this is unlikely given the stature and nature of the HTA programme, further work would be needed to test this hypothesis.

## Conclusions

The PRO components of HTA clinical trial protocols require improvement. Detailed instructions on the PRO rationale/hypothesis, data collection methods, training and management were often absent from protocols, even where the PRO was the primary outcome. This low compliance is unsurprising as existing PRO guidance for protocol writers lacks consistency and is difficult to access, whilst PRO-specific protocol items are not fully addressed by the current SPIRIT statement. There is a need for consensus-based supplementary guidelines outlining recommended standard PRO content for inclusion within trial protocols.

## Supporting Information

Appendix S1 **Full PRO protocol checklist.**
(XLSX)

Appendix S2 **Full list of PROMs used across included protocols.**
(XLSX)

Appendix S3 **Full multiple regression model.**
(XLSX)

Checklist S1 **PRISMA Checklist.**
(DOC)
